# Professor Forget on Interrogation of Patients

**Published:** 1861-04

**Authors:** 


### PROF. FORGET ON INTERROGATION OF PATIENTS.

This is surely a most fruitful source of error. Unless the practitioner be acute, the patient will almost always deceive. He deceives him through stupidity, ignorance or carelessness, as we often see among the people of the lower orders who are unintelligent and inattentive to matters relating to health. They always seem to strive to conceal the real trouble; they always assign senseless causes, and commit egregious errors concerning the dates of the invasion, the symptoms, etc. The patient deceives through prejudice, dissimulation, simulation, exaggeration, according to his material or moral interests. In the hospitals, especially, these causes of error abound, and they are common in society too. Every patient has a theory on the causes of his disease. Discreditable causes are always kept back. How often have you seen me establish conclusively the existence of a venereal disorder or of a pregnancy the patient has positively denied! Only the other day, a young man with a great mass of hemorrhoidal tumors insisted that he had no trouble about the anus. When inspection had revealed the real state of things, he excused himself on the ground that he was ashamed to admit such a disease. A woman whose genitals were studded with vegetations declared there was nothing the matter in this region. Simulation is, in all ranks of society, one of the traps against which the physician has great need to be on his guard. Exaggeration is common in others besides hypochondriacs. Patients deceive through bashfulness, through vanity, through the love of the marvellous, even through gratuitous mendacity. Rachitis and scrofulous ulcers are always the results of some accident encountered in childhood. A cicatrix, a fistulous sore has been produced by a glorious wound. Many patients task their powers in inventing extraordinary causes, symptoms, and therapeutic affects: one has an attack of pneumonia from eating a joint of mutton, another has dejections which scorch his linen, a third experiences formidable accidents from drinking flaxseed tea. Lastly, there are patients who take a malicious pleasure in deceiving their doctor; hysterical women especially manifest this aberration. In short the patient deceives the physician in every way and on every subject. It is a Herculean labor to criticise, curtail, and rectify the interrogation, not only in cases of ignorant people, but of those in the highest social position, for the latter have oftentimes the most ridiculous notions and the most rooted prejudices on all medical subjects. Hence it is that practitioners soon throw overboard those formal protocols, those tedious formularies of interrogation laboriously drawn up by systematic writers, and trust to little beyond the simple evidence of their own senses. This is what I call *veterinary.—Gazette Medicale.—Md. and Va. Journal.*

